# The Impact of Incomplete Faces of Spokes-Characters in Mobile Application Icon Designs on Brand Evaluations

**DOI:** 10.3389/fpsyg.2020.01495

**Published:** 2020-07-31

**Authors:** Zhang Ning, Liu Chunqun, Tong Zelin, Zhou Nan, Hu Yiting

**Affiliations:** ^1^College of Management, Shenzhen University, Shenzhen, China; ^2^College of Management, Hainan University, Haikou, China

**Keywords:** brand evaluations, incomplete spokes-character faces, perceptions of anthropomorphism, interpersonal closeness, social exclusion

## Abstract

In this article, we explore how incomplete spokes-character faces (versus complete spokes-character faces in application icon designs) make a positive impression on users, and we outline the boundary conditions. Across three studies, we find incomplete spokes-character faces to be an effective image icon tool. In study 1, we find that spokes-characters with incomplete faces improve users’ brand evaluations. In study 2, we find that incomplete spokes-character faces create perceptions of anthropomorphism, which lead to more favorable brand evaluations by enhancing the interpersonal closeness between the user and the brand. The results of study 3, however, show that the type of social exclusion (control vs. ignored vs. rejected) moderates the relationship between incomplete spokes-character faces in mobile application icons and brand evaluations.

## Introduction

Modern life has become more mechanized, automated and digitized, increasing convenience but also resulting in a loss of a sense of humanity (Schroll et al., 2018). Because human connection is a basic need for individuals (Baumeister and Leary, 1995), many managers aim to personalize their brands, products or spokes-characters by giving them human characteristics (Garretson and Burton, 2005; Aggarwal and Mcgill, 2012; Folse and Burton, 2012; Nazuk and Sajeev, 2018) to achieve brand differentiation.

Notably, marketers make full use of spokes-character strategies in mobile applications with the increasing popularity of mobile Internet. Currently, marketers often apply spokes-characters in icon designs for mobile applications to improve the liveliness of pages. In particular, spokes-characters are used in the designs of launch icons of apps. A spokes-character can act as the “face” of an app, which leaves an impression on users when they learn about the app in an ad or use it in daily life. The app launch icon (hereafter referred to as the icon) can be seen in app store lists, where the app can be downloaded, and can be clicked to access the app; in addition, the spokes-character faces on these icons can be manipulated. Interestingly, some firms (e.g., Hopster; see Appendix A) use spokes-characters with incomplete faces in icon designs, which we refer to as incomplete spokes-character faces, whereas other companies (e.g., Happy Cow Find Vegan Food; see Appendix A) use complete spokes-character faces in icon designs. However, it is not clear whether these incomplete spokes-character faces in icons truly enhance perceptions of anthropomorphism or generate any positive effects. Our primary research questions are whether the use of such incomplete spokes-character faces affect brand evaluations and, if so, how.

In previous studies, Hagtvedt (2011) shows that incompleteness firm’s name (the characters of company’s name are left intentionally blank. e.g., IBM) improves perceptions of a firm’s innovativeness and lowers perceptions of its trustworthiness. Nazuk and Sajeev (2018) explored the effect of AWS (Active White Space: the space between individual logo design elements—as a modification of the logo design but still retain its existing associated style modification. e.g., the logo of Starbucks image) on pictorial logo designs. However, our work differs from Hagtvedt (2011) in that their work were limited to typeface logos or brand logo designs with intentionally blank, whereas we investigate the effect of the incomplete spokes-character in the mobile icon that the face of the spokes-character seems to be intentionally hidden (e.g., Happy Cow Find Vegan Food; see Appendix A) and interact with users.

In response, this article offers a systematic investigation of the effects of the use of incomplete spokes-character faces in mobile application launch icons on brand evaluations. The facial completeness of spokes-characters in mobile application launch icon designs is easy to manipulate, and the launch icon often is the first touchpoint users have with a mobile application. Thus, in study 1, we start by demonstrating a positive effect of the use of incomplete spokes-character faces in the design of mobile application launch icons. The next study reveals the serial mediation process underlying this effect (completeness → perceptions of anthropomorphism → interpersonal closeness → brand evaluations). Study 2 shows that using an incomplete (vs. complete) spokes-character face increases perceptions of anthropomorphism, which lead to more positive brand evaluations by enhancing users’ interpersonal closeness to the spokes-character. Finally, in study 3, we demonstrate that the opposite results are found when the individual is rejected than when the individual is ignored.

These findings contribute to several research streams. First, based on gestalt theory, some scholars believe that incomplete objects (e.g., product advertisements, product names, ad photos, and brand logos) may make individuals seek the closure of the designs (Nazuk and Sajeev, 2018), which may make impressions on them in the process and result in positive evaluations (Peracchio and Meyers-Levy, 1994; Henderson and Cote, 1998; Miller and Kahn, 2005; Pieters et al., 2010; Hagtvedt, 2011; Nazuk and Sajeev, 2018). In our research, visual metaphor theory illustrates why individuals positively evaluate incomplete spokes-character faces. Considering spokes-characters with anthropomorphic characteristics, we assume that when a spokes-character has an incomplete face in an icon, users may believe that the spokes-character is “playing” with them similar to the way a human would, which may make users feel closer to the spokes-characters and assess the brand positively.

Second, our idea that an incomplete spokes-character face humanizes an animal by creating perceptions of anthropomorphism is related to but different from previous studies on anthropomorphism. Anthropomorphism refers to the attribution of human-like qualities to non-human objects (Aggarwal and Mcgill, 2007; Epley et al., 2007) by making a product appear alive (Waytz et al., 2010). Although the tendency to anthropomorphize is pervasive, people do not anthropomorphize all objects (Guthrie, 1993), nor are they able to anthropomorphize different objects with equal ease. The literature suggests that the ability to anthropomorphize may depend on the presence of specific features (Tremoulet and Feldman, 2000). For example, movement in an object (a spokes-character with an incomplete face) can create the impression that it is alive (Tremoulet and Feldman, 2000). We extend the scope of anthropomorphism theory. This broader perspective not only enhances the theoretical understanding of humanization processes but also provides a novel way for companies to endow their mobile application icons with anthropomorphic features.

Third, previous studies on spokes-characters have focused on the effects of its characteristic (e.g., sincerity, excitement, and competence, Callcott and Alvey, 1991) on building brand equity (Folse and Burton, 2012; En-Chi, 2014) or on brand-defending (Aaker et al., 2004; Folse et al., 2013). However, there is a lack of research on the shape characteristics of spokes-characters. In the present research, we emphasize the impact of incompleteness in the design of spokes-characters’ faces in icons on the perceptions of anthropomorphism and users’ perceptions of interpersonal closeness between users and spokes-characters, which benefits brand evaluations.

## Literature Review

### Incomplete Spokes-Character Faces

Spokes-characters, often referred to as advertising icons (visual images that are cartoon- or human-like), offer potential benefits to brand equity and can symbolically communicate a brand’s attributes, personality, or benefits (Garretson and Burton, 2005; Folse and Burton, 2012). Most research on spokes-characters concerns brand building, such as establishing unique and favorable brand evaluations (Barbara, 1996; Phillips, 1996). In detail, the characteristics (e.g., likability, expertise, and attractiveness) help shape brand perceptions (Callcott and Alvey, 1991) or brand equity (Folse and Burton, 2012; En-Chi, 2014). In addition, a few scholars have studied the role of spokes-characters’ personality traits in brand defense (Aaker et al., 2004; Folse et al., 2013).

Most scholars have focused on the effect of the personality of the spokes-character on brand evaluations. However, few studies have explored how to design spokes-characters in specific situations, such as in icons for mobile applications or web pages. We find that there are two kinds of spokes-character designs in icons: those with incomplete faces and those with complete faces. Previous studies have provided some insight into completeness (e.g., in sculptures, brand names, and brand logos). Studies have shown that incomplete objects prompt people to seek closure or provide explanations for missing parts by making them search for missing parts, which leads to positive product evaluations (Peracchio and Meyers-Levy, 1994). Moreover and Hagtvedt (2011) indicates that incompleteness in brand name designs (when the letters in the name are intentionally left blank, such as the logo of IBM) induces consumer perceive the company more innovativeness and lowers trustworthiness and this influence is mediated by the perceived interestingness of the incomplete brand name compared with the complete one. In contrast, consumers perceive the complete brand names to be more reliable and less innovative and this effect is mediated by the perceived clarity of the name (Hagtvedt, 2011).

Generally speaking, the signal may be a perceptible stimulus. Literatures on social signal processing indicate that an individual’s sensory device receives a physical stimulus (a pattern of physical energy), and dynamically models the influence of Gestalt’s law (Wertheimer, 1938). In addition, the signal may be developed by a virtual character, an animal, a machine or other entities (Poggi and D’Errico, 2010a). In particular, the signal is an information (a simple or complex perception produced by one or more physical stimuli), from which the receiver can extract more information (Vinciarelli et al., 2011). Therefore, the spokes-character in the icon, a kind of signal, also can make users gain information and develop perceptions to the icon or the brand. Some scholars propose that compared with a complete logo, an incomplete logo can promote consumers to make an effort to comprehend the logo, which makes the brand communication information clearer and leads to more positive visual evaluations (Nazuk and Sajeev, 2018). A complete entity makes people feel comfortable in terms of the visual balance (Pracejus et al., 2006; Olsen et al., 2012). In addition, some studies have shown that perceptual ambiguity caused by incomplete objects prompts people to seek closure or provide explanations for missing, which can improve evaluations by stimulating positive emotions (Peracchio and Meyers-Levy, 1994). In providing the missing visual portions themselves, individuals would induce the sense of accomplishment when they resolve the slight visual ambiguity, which increases the overall positive affect derived in the process (Peracchio and Meyers-Levy, 1994; Nazuk and Sajeev, 2018). Similarly, an incomplete object (e.g., a logo or name) may prompt people to seek closure for the missing part, thereby inducing positive attitude after that (Peracchio and Meyers-Levy, 1994; Zhao and Meyer, 2007; Nazuk and Sajeev, 2018).

Based on conceptual metaphor theory, our research speculates that when spokes-characters are featured in an icon design, the user will focus on providing explanations for a spokes-character with an incomplete face (the spoke-character is playing with users) rather than try to imagine the complete face of the spokes-character (seeking closure). Metaphor is the basis of people’s cognition, thinking, experience, language and even behavior. It is the mapping composition in different experience fields or conceptual structures. Metaphor theory emphasizes the similarity between ontology objects and metaphor objects (Lakoff and Mark, 1980). Visual metaphor is a type of metaphor that indicates that an object is similar to another object by comparing two completely different images (Lagerwerf et al., 2012) and that provides an effective way to understand new things through the transplantation of elements between unfamiliar and familiar things (Zhong and Zhang, 2009). Previous research has shown that visual metaphors cannot be fully described in formal terms. Instead, they must be viewed as visual representations of metaphorical ideas or concepts (Refaie, 2003). Visual metaphor is a kind of metaphor. We define visual metaphor as a conceptual metaphor, which is caused by visual stimuli and causes personal thinking. Individuals tend to make comparisons with sentimentally similar objects, which are likely to be based on size, shape, spatial orientation, color, etc. (Schilperoord et al., 2009). Moreover, visual metaphors include four aspects: (1) natural phenomena, (2) artificial reality, (3) activities, and (4) abstract concepts (Eppler and Burkard, 2004). Visual metaphor involves physical similarity (e.g., between an egg and the earth) or psychological similarity (e.g., between a dove and peace).

What is more, the signal may be a perceptible stimulus. Literatures on social signal processing indicate that an individual’s sensory device receives a physical stimulus (a pattern of physical energy), and dynamically models the influence of Gestalt’s law (Wertheimer, 1938). In addition, the signal may be developed by a virtual character, an animal, a machine or other entities (Poggi and D’Errico, 2010a). In particular, the signal is an information (a simple or complex perception produced by one or more physical stimuli), from which the receiver can extract more information (Vinciarelli et al., 2011). Therefore, the spokes-character in the icon, a kind of signal, also can make users gain information and develop perceptions to the icon or the brand. Besides, social emotions refer to those emotions related to social relations (Lewis, 2000), such as pride, shame or embarrassed, or feelings of disgust, jealousy, contempt, admiration to others (Rizzi, 2007; Poggi and Zuccaro, 2008). The expression of some social emotions is social signals (e.g., interest, empathy, hostility, agreement, dominance, superiority, etc.) as these emotions indicate a specific relationship with others (Vinciarelli et al., 2011). Social exclusion will make individuals generate negative social emotions, which leads to them convey negative social signal to others. We suggest that an incomplete spokes-character face has similar characteristics to the person who is playing hide-and-seek game with us. When a spokes-character’s face is incomplete, the individual will regard the spokes-character as a person who seems to be playing with him or her. That is, spokes-characters with incomplete faces visually appear to be engaging in an activity similar to a real human.

Prior advertising research has shown that visual metaphors with moderate complexity have more positive effects on evaluations than simpler or much more complex visual metaphors (Mulken et al., 2014). Similarly, compared with a complete spokes-character face (no visual metaphor) in an icon, we expect incomplete spokes-character face (a moderately complex visual metaphor) to have a much more positive impact on brand evaluations. Therefore, we expect that incomplete spokes-character faces in icons can make users develop more positive brand evaluations than complete spokes-character faces. Accordingly, we propose the following hypothesis:

H1: Adopting an incomplete (vs. complete) spokes-character face in a mobile application icon will lead to more positive brand evaluations.

### Anthropomorphism and Interpersonal Closeness

Anthropomorphism refers to the attribution of human characteristics to non-human entities (Guthrie, 1993; Epley et al., 2007, 2008). Prior works have shown that anthropomorphism using visual or linguistic portrayals can evoke a human schema (Guthrie, 1993; Epley et al., 2007, 2008), which promotes consumers’ perceptions of the product as having human-like characteristics (Aggarwal and Mcgill, 2007). Previous studies on incomplete aesthetic works, brand names and brand logos are different from those on incomplete spokes-character faces. The former studies focused on the cognition of individuals when they see complete incomplete images or typefaces (Peracchio and Meyers-Levy, 1994; Zhao and Meyer, 2007; Nazuk and Sajeev, 2018). Scholars put forward that incompleteness prompts individuals to complete the entity and make it completed, thus bringing a positive attitude. However, we argue that the findings of the latter studies reflect the positive effects of anthropomorphized spokes-characters. The literature suggests that the ability to anthropomorphize may depend on the presence of specific features (Guthrie, 1993). One of the motivations for people to anthropomorphize objects is that they can make better sense of the environment around them (Guthrie, 1993). Individuals make use of what they have already mastered or have become familiar with to help them understand the things that they know less about by attributing human-like characteristics to objects (Aggarwal and Mcgill, 2007). When the company uses an incomplete spokes-character face in an icon, users may view the spokes-character as playing with them similar to the way a person would to explain why the spokes-character has an incomplete face.

Furthermore, not all objects can give rise to anthropomorphic perceptions. If an object can move, it may make an individual perceive that it is alive (Tremoulet and Feldman, 2000). However, an object that moves quite slowly (e.g., clocks) may seem to lack human characteristics in this aspect (Morewedge et al., 2004). Moreover, some scholars have proposed that a static logo can lead to the perception of movement, which may enhance consumer engagement and attitudes (Cian et al., 2014). In our study, a spokes-character with an incomplete face (a static image) in an icon design can also make users feel that the spokes-character appears to be moving because it creates the illusion that it is “playing” with users, which may promote a perception of movement and prompt individuals to anthropomorphize the spokes-character. In addition, the literature suggests that pictures in advertising, which creates a metaphor for a product that appears to be engaged in some type of human behavior, may result in perceptions of anthropomorphism and generate greater brand preferences (Delbaere et al., 2011). Therefore, in our study, we assume that using an incomplete spokes-character face in an icon may generate user perceptions of the movement and anthropomorphism of the spokes-character.

In our research, based on visual metaphor theory, we believe that using an incomplete (vs. complete) spokes-character face in a launch icon will result in higher degrees of anthropomorphism of the spokes-character as well as users’ perceptions of interpersonal closeness, which leads to the development of positive brand evaluations. Interpersonal closeness is a dimension of interpersonal communication (Burgoon and Hale, 1987) that refers to the perception of interpersonal distance during interpersonal communication. In our study, interpersonal closeness refers to the perception of closeness between users and the spokes-character. If a person perceives closeness with another person, he or she may be more likely to develop trust, communication and intimacy with the other person (Woosnam, 2010). However, regarding users, the level of closeness perceived with spokes-characters in icons is quite difficult for companies.

In addition, a spokes-character is not only a brand identification symbol but also an important way for brands to establish relations with consumers (Callcott and Phillips, 1996). The incomplete spokes-character as a virtual character convey social emotions to users. The expression of some social emotions is social signals (e.g., interest, empathy, hostility, interactivity, etc.) as these emotions indicate a specific relationship with others (Vinciarelli et al., 2011).

Therefore, when a company uses an incomplete spokes-character face in an icon, users of the mobile application will perceive the spokes-character to be dynamic. It will appear that the spokes-character is playing with users. That is, a spokes-character with an incomplete face makes users develop closer interpersonal perceptions with it by enhancing their perceptions of anthropomorphism, which will lead them to develop more positive brand evaluations. Therefore, we offer a second hypothesis:

H2: Adopting an incomplete (vs. complete) spokes-character face in designing a mobile application icon will enhance users’ perceptions of anthropomorphism, which will lead to more favorable brand evaluations by enhancing interpersonal closeness.

### Social Exclusion

Social exclusion is a common phenomenon (Mazzini et al., 2011). For instance, Leary et al. (2003) found that the principals of the 13 shootings have all been rejected in 15 school shootings from 1995 to 2001, and that there may be a close connection between the rejection and the attack. As a kind of social animal, human beings must rely on groups to obtain better opportunities for survival, reproduction and development (Su et al., 2017). If an individual is rejected by others, its life will be seriously threatened. Social exclusion has two forms: being ignored and being rejected (Molden et al., 2009; Lee and Shrum, 2012; Sinha and Fang-Chi, 2019). Social neglect refers to when an individual is ignored by others or excluded from a group in social communication, which develops negative emotions or evaluations (Williams, 2007). Social rejection refers to an individual being specifically excluded and negatively evaluated by others or groups, which leads to strong negative emotional experiences (Leary et al., 2003). Individuals who suffer from social rejection may clearly feel the rejection of others and believe that they are not liked, which can even make them adopt revenge behaviors, such as reducing pro-social behaviors (Twenge et al., 2007). However, when individuals are ignored by others, they will engage in pro-social behaviors (Dewall et al., 2007). In addition, empirical studies have compared the effects of different types of exclusion on individuals. These studies have shown that rejected individuals have stronger preventive motivation. Rejected individuals may avoid social interaction and try to avoid behaviors that may lead to exclusion. Those who are ignored have stronger primitive motives. To form interpersonal connections with others, they actively participate in social interactions and consider what measures can be taken to avoid being excluded (Molden et al., 2009; Su et al., 2017). These findings suggest that different types of social exclusion elicit different responses. The exclusion of an individual sometimes is accompanied by explicit antipathy from other people but sometimes is not (Twenge et al., 2001; Lee and Shrum, 2012).

In our work, we propose that individuals who are ignored by others will develop positive brand attitudes toward a company that uses an incomplete spokes-character face in an icon. Existing empirical studies have compared the impact of different types of exclusion on individuals, and found that rejected people will have stronger prevention motivations (prevention motivations), avoiding social interactions, pay attention to avoid behaviors that may lead to exclusion; the neglected people have stronger promotion motivations, actively participate in social interactions, and consider what measures can be taken to avoid exclusion (Molden et al., 2009). Some scholars have reported that individuals who are treated with pro-social behavior have more positive emotions toward and evaluations of the group and others. In this way, their basic needs for belonging are satisfied (Williams, 2007). Therefore, pro-social behavior not only benefits the recipient but also enables the individual who shows pro-social behavior to obtain more positive social evaluations (Clary et al., 1998) and more positive emotional experience (Aknin et al., 2015). We suggest that a spokes-character with an incomplete face will make individuals who are being ignored feel that the spokes-character is showing pro-social behavior toward them and that they will evaluate the brand more positively than a brand with a complete spokes-character face.

In addition, we suspect that the individuals who are being rejected will negatively assess a brand that uses a spokes-character with an incomplete face in its icon design. Studies have found that social exclusion triggers individuals’ negative emotions and negative influences individuals’ self-evaluations (Baumeister et al., 2005), such as bitterness. The bitterness is a negative emotion between anger and sadness. It is usually caused by betrayal, which comes from the disappointment of the emotional expectations of oneself or others (Poggi and D’Errico, 2010b). This negative emotion affects individuals’ social behaviors (Twenge et al., 2002). The expression of some social emotions is social signals as these emotions indicate a specific relationship with others (Vinciarelli et al., 2011). Previous research has shown that participants with anticipated regret forces would chose the safe entity, avoiding risk-aversion (Zeelenberg et al., 1996). So we believe that even when a company’s icon design uses a spokes-character with an incomplete face, people who are rejected would not have a need to form relationships with other objects and they will lack motivation to personify objects, which make them withdrawal from social contact (Twenge et al., 2002, Twenge et al., 2007). Hence, they can not feel the social signal from the spokes-character with incomplete face in the icon. To the opposite, the individual who are ignored by others would increase social sensitivity and renewed efforts toward social connection and they can realize the social signal from the spokes-character with incomplete face in the icon.

The reason why people tend to anthropomorphize objects is that doing so may comfort them by providing relationships or companionship (Guthrie, 1993). Individuals who are rejected may develop preventive motivation, which causes people to avoid social contact to avoid the possibility of experiencing a lack of belonging (Molden et al., 2009). Therefore, we believe that even when a company’s icon design uses a spokes-character with an incomplete face, people who are rejected will not have a need to form relationships with other objects. Hence, they will lack motivation to personify objects, so they will not feel interpersonal closeness with the spokes-character. Accordingly, we hypothesize the following:

H3a: When an individual is rejected, the use of an incomplete (vs. complete) spokes-character face in a mobile application icon design will reduce users’ perceptions of interpersonal closeness, which will lead to more negative brand evaluations.

H3b: When an individual is ignored, the use of an incomplete (vs. complete) spokes-character face in a mobile application icon design will enhance users’ perceptions of interpersonal closeness, which will lead to more positive brand evaluations.

## Study

### Study 1

In experiment 1, we manipulated an incomplete spokes-character face to test the effect of the incomplete spokes-character face on brand evaluations. We predicted that participants would have more positive brand evaluations when the icon design featured an incomplete spokes-character face than when it featured a complete spokes-character face. In study 1, we employed a one-factor (completeness: incomplete vs. complete) between-subjects design.

#### Procedure

First, we introduced a fictional clothing brand called “HO” to the participants. Then, we told them that “Little H” was the spokes-character of “HO” and presented participants with one of two versions of the mobile application icon images (see Appendix B) that included either a complete or an incomplete face of “Little H.” After that, the participants were asked to evaluate the facial completeness of “Little H” (1 = incomplete, 7 = complete). Subsequently, we also measured how the participants evaluated the brand with four seven-point scales: “Please evaluate this brand on the following dimensions: dislike/like, bad/good, unappealing/appealing, and unfavorable/favorable” (α = 0.87) (Puzakova and Aggarwal, 2018). In addition, the subjects were asked to report their emotions with four items: not at all happy/very happy; not at all happy/very happy; not at all excited/very excited; not at all hopeful/very hopeful; in a bad mood/in a good mood; not at all excited/very excited; not at all hopeful/very hopeful; in a bad mood/in a good mood (α = 0.86) (Hagtvedt, 2011). All scales used a 7-point scale (1 = strongly disagree and 7 = strongly agree). Finally, we collected the demographic information and completed the experiment.

#### Pretest

Prior to the main experiment, 52 undergraduate students (32 females, *M*_*age*_ = 20.04, *SD*_*age*_ = 0.97) from Shenzhen University in China participated in our study. They were randomly assigned to two groups (completeness: incomplete vs. complete). Then, we showed one of two icons (see Appendix B) and asked them to evaluate the completeness of the spokes-character’s face in the icons. In addition, to test whether there was a difference in the perceived cuteness between the two groups, the participants reported the likability (α = 0.92) of the spokes-character (Callcott and Alvey, 1991). Finally, the participants provided information about their gender and age.

To test whether the manipulation of the facial completeness of the spokes-character in the icon was successful, we conducted a one-way ANOVA with completeness as the dependent variable. The results showed that there was a significant difference between the two groups [*F*(1,50) = 30.52, *p* < 0.001]. The participants in the complete group indicated a higher degree of completeness (*M*_*C*_ = 6.22, *SD* = 0.75) higher than those in the incomplete group (*M*_*IC*_ = 4.60, *SD* = 1.20). In addition, the spokes-character with an incomplete face was not considered to be cuter (*M*_*IC*_ = 4.71, *SD* = 1.22) than the spokes-character with an incomplete face [*M*_*C*_ = 4.82, *SD* = 0.75; *F*(1,50) = 0.13, *p* = 0.72], indicating that the effect was not driven by the perceived cuteness of each design.

#### Method

One hundred participants were recruited from a Chinese online platform^1^ that is similar to MTurk Prime. The platform provides a large subject pool with qualified participants. Four participants who failed to complete the survey were excluded from the final analyses. The remaining 96 participants provided complete datasets (71 females; *M*_*age*_ = 24.59, *SD*_*age*_ = 8.22).

#### Results and Discussion

##### Manipulation check

We conducted a one-way ANOVA of users’ perceptions of the facial completeness of the spokes-characters. The results showed that the participants in the complete condition rated the face as more complete (*M*_*C*_ = 5.67, *SD* = 1.16) than those in the incomplete condition [*M*_*IC*_ = 4.18, *SD* = 1.16, *F*(1,95) = 40.53, *p* < 0.001], confirming that our manipulation was successful.

##### Brand evaluations

The results of the one-way ANOVA of brand evaluations showed that the main effect of the facial completeness of spokes-character was significant. The participants the incomplete spokes-character face condition reported more positive brand evaluations (*M*_*IC*_ = 5.04, *SD* = 1.02) than those in the complete spokes-character face condition [*M*_*C*_ = 4.21, *SD* = 1.00, *F*(1,95) = 16.01, *p* < 0.001]. Therefore, compared with an complete spokes-character face in an icon design, an incomplete spokes-character face leads to more positive brand evaluations, which verifies hypothesis 1.

##### Control variables

Furthermore, an incomplete spokes-character face might affect perceptions of the spokes-character’s personality. We examined the emotions of the participants. The data indicated that the completeness of the spokes-characters’ faces affected the participants’ emotions [*M*_*IC*_ = 4.88, *SD* = 0.89; *M*_*C*_ = 4.19, *SD* = 126, *F*(1,95) = 9.68, *p* < 0.01]. However, when we controlled for emotion, a positive effect of the incomplete spokes-character face still existed.

Study 1 provided evidence of our main prediction: using a spokes-character with an incomplete (vs. complete) face in a launch icon positively affects brand evaluations. In the next study, we tested the mechanism of the positive effect of an incomplete spokes-character face in an icon design on brand evaluations with a mediation analysis.

### Study 2

In this study, we measured the perceived anthropomorphic and interpersonal closeness of the spokes-character to test the underlying mechanism of the positive effect. We investigated whether the use of an incomplete (vs. complete) spokes-character face in a launch icon enhanced perceptions of anthropomorphism and in turn increased participants’ perceptions of interpersonal closeness to the spokes-character to ultimately lead to more favorable brand evaluations.

#### Design

We employed a single-factor (completeness: incomplete vs. complete) between-subjects design. One hundred thirty participants with various backgrounds were recruited from Sojump^1^. Two participants who failed to complete the survey were excluded from the final analyses. The remaining 128 participants provided complete datasets (68 females; *M*_*age*_ = 29.29, *SD*_*age*_ = 7.25). We used the download interface of a mobile application for a fictitious snack brand (Bear Snack) as our stimulus to extend our findings (see Appendix C).

#### Procedures

We introduced there was a fictitious snack APP and show the interface of the application to participants. After that, the participants answered the same manipulation item as in study 1. Then, we asked the participants to evaluate the facial completeness of the spokes-character (α = 0.88). Next, we measured perceptions of anthropomorphism with three items (“It seems almost as if the spokes-character has a mind of its own”; “To what extent does the spokes-character remind you of some human-like qualities?”; “The spokes-character looks like a person”) (1 = “not at all” and 7 = “very much”) (α = 0.77) (Puzakova et al., 2013; Hur et al., 2015; Puzakova and Kwak, 2017) and interpersonal closeness with three items (including “I feel so closed to the spokes-character” “There is a similarity between me and the spokes-character” and the Inclusion of Other in the Self Scale (IOS) adopted from prior research on interpersonal evaluations (α = 0.79) (Aron et al., 2000; Orehek et al., 2018). Finally, the participants provided brief demographic information.

#### Results and Discussion

##### Manipulation check

We conducted a manipulation check on facial completeness and demonstrated that users in the complete face condition perceived the facial completeness of the spokes-character to be more complete than those in the incomplete face condition [*M*_*C*_ = 6.03, *SD* = 1.04; *M*_*IC*_ = 3.95, *SD* = 1.50; *F*(1,127) = 83.40, *p* < 0.001].

##### Brand evaluations

We analyzed our predictions using a one-factor (completeness: incomplete vs. complete) ANOVA with brand evaluations as the dependent variable. The results revealed significant differences in brand evaluations [*M*_*IC*_ = 5.60, *SD* = 0.87; *M*_*C*_ = 4.99, *SD* = 1.014; *F*(1,127) = 13.14, *p* < 0.001], which supported H1.

##### Serial mediation analyses

**FIGURE 1:**
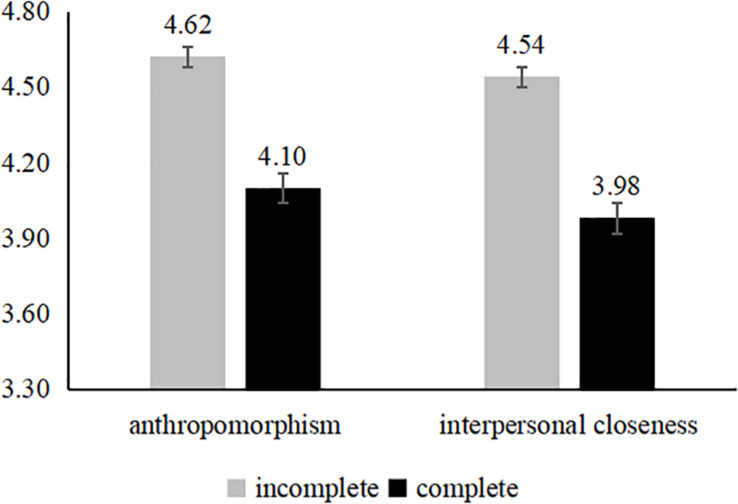
Mediation analyses of study 2.

The data indicates (see Figure 1) that compared with the complete group, the group of the spokes-character with incomplete face in the icon cause more positive perceptions of anthropomorphism [*M*_*IC*_ = 4.62, *SD* = 1.21; *M*_*C*_ = 4.10, *SD* = 1.09; *F*(1,127) = 10.33, *p* < 0.001; see Figure 1] and interpersonal closeness [*M*_*IC*_ = 4.54, *SD* = 1.32; *M*_*C*_ = 3.98, *SD* = 1.25; *F*(1,127) = 15.21, *p* < 0.001]. To further examine the underlying mechanism of the effect of an incomplete spokes-character face in an icon design, we tested regression models with incomplete spokes-character face, perceptions of anthropomorphism, and interpersonal closeness as the mediators and brand evaluations as the dependent variable (Hayes, 2013, Model 6) using a bootstrapping approach. Consistent with H2, we found that perceptions of anthropomorphism and interpersonal closeness mediated the effect of incomplete spokes-character face on brand evaluations (95% CI [−0.0647, −0.0049]).

The results indicated that the positive impact of an incomplete spokes-character face is driven by enhanced perceptions of anthropomorphism and then by enhanced interpersonal closeness, supporting H2. That is to say, compared with the complete face spokes-character in the mobile icon, when users see the spokes-character with incomplete face, the perceptions of anthropomorphism and interpersonal closeness with the spoke-character would be more stronger, which induces more positive users evaluations. In the next study, we explored the boundary conditions.

### Study 3

#### Design

In the experiment, we employed a 2 (completeness: incomplete vs. complete) × 3 (social exclusion: rejected vs. ignored vs. control) between-subjects design. Four hundred and ten participants were recruited from a Chinese online panel^2^. Eight participants who failed to complete the survey were excluded from the final analyses. The remaining 402 participants provided complete datasets (255 females; *M*_*age*_ = 26.19, *SD*_*age*_ = 7.07).

#### Procedures

First, we manipulated the type of social exclusion. In the control condition, the participants were asked to recall something that happened to them yesterday and write it down. In the rejected condition, the participants were asked to recall an experience of being strongly excluded for a period of time and being told they were not accepted because of a strong dislike or other reasons, and then they were asked to write it down. In the ignored condition, we asked subjects to recall an experience of being strongly ignored in which no one told them they disliked them, but others ignored them or obviously ignored their responses, and then they were asked to record it. Next, the participants evaluated the degree of being ignored/rejected (Molden et al., 2009). Furthermore, after the manipulation, the subjects reported the extent to which they were ignored and rejected (Molden et al., 2009). Second, we presented the APP icons with different completeness and the manipulation and manipulation check for the facial completeness of the spokes-character in the icon design were similar to those in study 1. In order to present different stimulation scenes, the interface in this study was a download interface (see Appendix B). Subsequently, we presented the icon again and measured the participants’ brand evaluations as in study 1 and their perceptions of anthropomorphism and interpersonal closeness (Woosnam, 2010; Orehek et al., 2018). Finally, we collected the demographic information of the subjects.

#### Results

##### Manipulation check

First, we need to verify that there were significant differences among the three groups of subjects in the two manipulation check items for the types of social exclusion. We analyzed the degree of rejection and neglect under three conditions (rejected vs. ignored vs. control). The *post hoc* test (LSD test) showed that in the first question (the degree of being ignored), there were significant differences between the ignored condition (*M* = 5.14, *SD* = 1.70) and the control condition [*M* = 3.28, *SD* = 1.71; *F*(1,273) = 81.46, *p* < 0.001]. Additionally, the participants in the ignored condition reported a higher level of neglect (*M* = 5.14, *SD* = 1.70) than those in the rejected condition [*M* = 4.58, *SD* = 1.83; *F*(1,273) = 6.54, *p* < 0.05]. For the second question (the degree of being rejected), there were significant differences between the rejected group (*M* = 5.02, *SD* = 1.61) and the control group (*M* = 2.56, *SD* = 1.86) [*F*(1,266) = 134.84, *p* < 0.001]. Similarly, there was higher level of rejection in the rejected condition (*M* = 5.02, *SD* = 1.86) than in the ignored condition [*M* = 4.46, *SD* = 1.62; *F*(1,259) = 6.60, *p* < 0.05]. Thus, the manipulation of social exclusion was successful. Second, we also examined the manipulation of the facial completeness of the spokes-character. The results showed that the score of the complete condition was significantly higher than that of the incomplete condition [*M*_*C*_ = 5.36, *SD* = 1.43; *M*_*IC*_ = 4.33, *SD* = 1.53; *F*(1,400) = 48.39, *p* < 0.001]. Therefore, our manipulations were successful.

##### Brand evaluations

**FIGURE 2:**
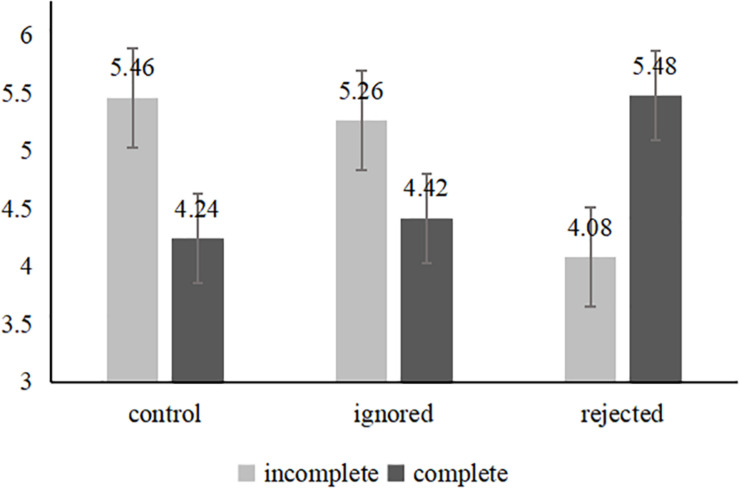
Brand evaluations in Study 3.

A two-way interaction showed that the main effect of the completeness of the spokes-character face on brand evaluations was not significant [*F*(1,401) = 3.36, *p* = 0.62], similar to the result for social exclusion [*F*(2,400) = 0.51, *p* = 0.60]. However, the interaction effect was significant [*F*(2,400) = 59.42, *p* < 0.001, see Figure 2]. In the control group, the participants evaluated the brand more positively (*M* = 5.46, *SD* = 0.85) when an incomplete spokes-character face was used than when a complete spokes-character face was used [*M* = 4.34, *SD* = 1.15; *F*(1,139) = 43.49, *p* < 0.001]; the same result was observed for the participants in the ignored group [*M*_*IC*_ = 5.26, *SD* = 1.00; *M*_*C*_ = 4.42, *SD* = 1.25; *F*(1,132) = 18.36, *p* < 0.001]. In contrast, the participants who were asked to recall being rejected reported more negative brand evaluations of the icon with the spokes-character with an incomplete face [*M*_*IC*_ = 4.08, *SD* = 1.07; *M*_*C*_ = 5.48, *SD* = 0.71; *F*(1,125) = 74.72, *p* < 0.001]. Therefore, when individuals are ignored, they have more positive brand evaluations when an incomplete spokes-character face is featured in an icon. However, when individuals are rejected, their evaluations of brands with complete spokes-character face become more negative. Moreover, there were no significant differences between the control group and the ignored group for either the incomplete spokes-character face [*M*_*C*_ = 5.26 vs. *M*_*IC*_ = 5.26; *F*(1,144) = 18, *p* = 1.74] or the complete spokes-character face [*M*_*C*_ = 4.34 vs. *M*_*IC*_ = 4.42; *F*(1,129) = 0.12, *p* = 0.73]. In addition, when the icon had an incomplete spokes-character face, the participants in the ignored group developed more positive attitudes toward the brand [*M*_*igored*_ = 4.34 vs. *M*_*rejected*_ = 4.42; *F*(1,131) = 42.84, *p* < 0.001] than those in the rejected group. Nevertheless, the participants in the rejected group preferred the icon with a complete spokes-character face more than those in the ignored group [*M*_*igored*_ = 4.42 vs. *M*_*rejected*_ = 5.48; *F*(1,126) = 34.06, *p* < 0.001].

##### Serial mediation analyses

**FIGURE 3:**
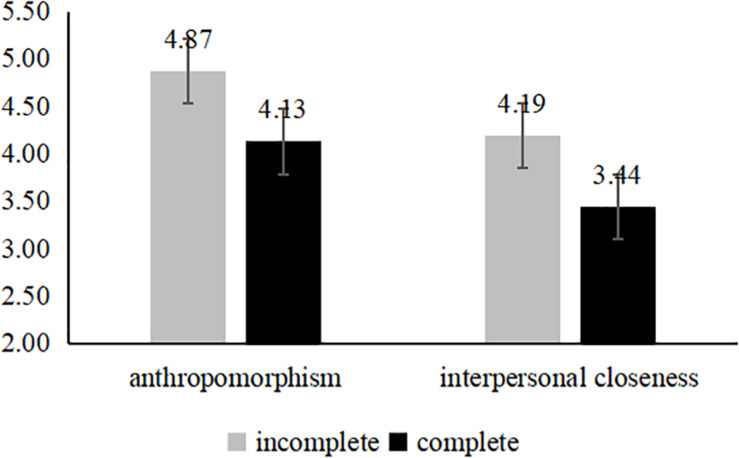
Mediation analyses of study 3 (the control group).

**FIGURE 4:**
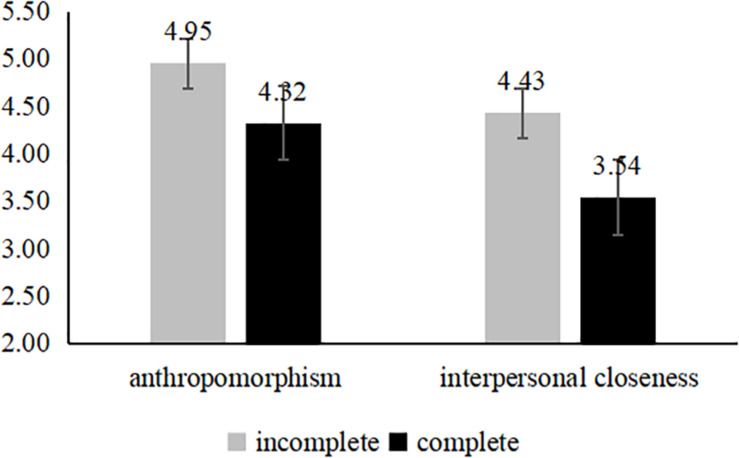
Mediation analyses of study 3 (the ignored group).

**FIGURE 5:**
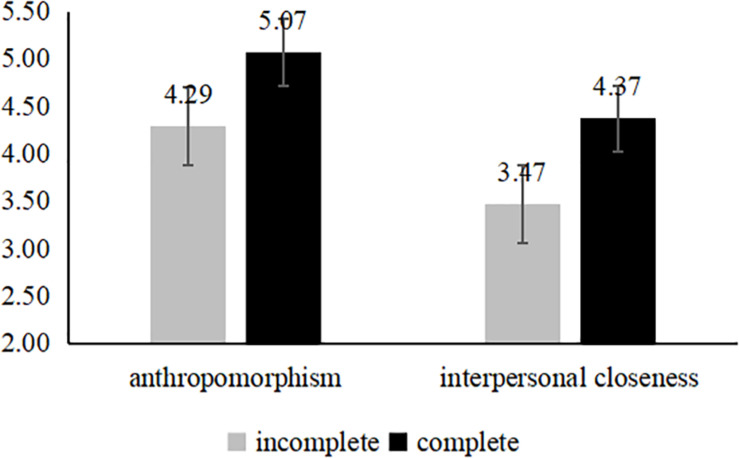
Mediation analyses of study 3 (the rejected group).

The data showed that: in the control group, the spokes-character with the incomplete face makes the perceptions of anthropomorphism [*M*_*IC*_ = 4.87, *SD* = 1.19; *M*_*C*_ = 4.13, *SD* = 1.28; *F*(1,139) = 12.79, *p* < 0.001] and interpersonal closeness [*M*_*IC*_ = 4.19, *SD* = 1.27; *M*_*C*_ = 3.44, *SD* = 1.18; *F*(1,139) = 13.12, *p* < 0.001] more stronger (see Figure 3). And the results the neglected group is same as to the control group (see Figure 4). However, for the rejected group, the complete face developed a higher level of the perceptions of anthropomorphism [*M*_*C*_ = 5.07, *SD* = 0.99; *M*_*IC*_ = 4.29, *SD* = 1.06; *F*(1,124) = 18.18, *p* < 0.001] and interpersonal closeness [*M*_*C*_ = 4.37, *SD* = 1.23; *M*_*IC*_ = 3.47, *SD* = 1.13; *F*(1,124) = 18.18, *p* < 0.001] (see Figure 5). We predicted that using an incomplete (vs. complete) spokes-character face in a mobile application icon would generate perceptions of anthropomorphism, which would lead to greater user perceptions of interpersonal closeness to the spokes-character and, ultimately, more positive brand evaluations (e.g., completeness → perceptions of anthropomorphism → interpersonal closeness → brand evaluations). To test this theoretical framework, we conducted a serial mediation analysis (Hayes, 2013, model 6, *n* = 5,000). The serial mediating effect was significant. In the ignored group, the mediating effect was significant and positive (95% CI [−0.2756, −0.0372], and the same result was observed for the control condition (95% CI [−0.2429, −0.0333]). In addition, in the rejected group, the mediating effect was significant (95% CI [0.0337, 0.1906]).

The results show that when the individual is ignored by other persons, the spokes-character in the icons with incomplete (vs. complete) face will induce more positive brand evaluations. Because the neglected persons are long for interpersonal relationships and are more sensitive to perceive the anthropomorphism of the spoke-character and social signals (interpersonal closeness) transmitted by incomplete faces, which leads to positive brand attitudes. In contrast, when the individual is rejected by others, the complete (vs. incomplete) face of the spokes-character in the APP icon will bring a more positive brand evaluation. The reason is the rejected individuals consciously avoid social interactions resulting generating resistance to living entity and social signals that transmitted by incomplete faces, which develops negative brand evaluations. Therefore, the results support hypotheses H3a and H3b.

## General Discussion

This study assessed the influence of incomplete spokes-character faces in mobile application icons on brand evaluations. Based on three experiments, we provide evidence for, reveal the mechanisms of, and outline the boundary conditions of the positive effect of incomplete spokes-character faces. In our first study, we show that using an incomplete spokes-character face in an icon enhances users’ brand evaluations. Study 2 reveals the underlying mechanisms: an incomplete spokes-character face leads to more favorable brand evaluations because the incompleteness humanizes the spokes-character by creating perceptions of anthropomorphism and thereby enhancing users’ perceptions of interpersonal closeness to the spokes-character. Moreover, we demonstrate that the effect is reversed for individuals who are being rejected (study 3).

There are some limitations to our research. In the future, scholars can conduct in-depth research on how to design an icon to increase the anthropomorphic perceptions of the spokes-character. First, we extend the humanization literature by introducing a spokes-character strategy in icon design rather than relying on previous research on anthropomorphism theory (Epley et al., 2007; Puzakova et al., 2013). That is, a spokes-character might be humanized by showing an unusual face, which may be a way to help users remember the spokes-character for a long time (Chiu et al., 2009). Our study shows that an incomplete spokes-character face can also provide humanization cues. Moreover, future research can explore movement of the spokes-character in an icon, which might enhance anthropomorphic perceptions (Calvert, 2008). Second, we contribute to the literature on incomplete objects, such as product names (Miller and Kahn, 2005), ad photos (Peracchio and Meyers-Levy, 1994), and logos (Henderson and Cote, 1998; Hagtvedt, 2011; Nazuk and Sajeev, 2018). Our research shows the effect of an incomplete spokes-character face in a mobile application icon on users’ brand evaluations. Future research can explore the effect in product packaging or advertisement design.

This research introduces a novel way for companies to humanize their brands which applies the incomplete spokes-character faces in mobile application icons. The current study further shows that the use of an incomplete spokes-character face improves brand evaluations through perceptions of anthropomorphism, which enhance users’ perceptions of interpersonal closeness to the spokes-character. However, the positive effect of an incomplete spokes-character face is less pronounced when users are rejected by others. In summary, this research serves as a foundation for examining humanization efforts in marketing communication that go beyond the use of anthropomorphism. We hope that further research will explore the moderation of brand personality (Aaker, 1997). Incomplete (vs. complete) spokes-character faces may lead users to perceive spokes-characters to be more exciting. According to perceptual fluency theory (Lee and Labroo, 2004; Nazuk and Sajeev, 2018), if a brand positions its personality as exciting and reflects this excitement with an incomplete spokes-character face in an icon, consumers can perceive this excitement in advertising and process it easily, which is good for brand communication. What’s more, social exclusion induces powerful motivations and a variety of negative emotions (e.g., sadness and fear) (Williams, 2007; Molden et al., 2009). Therefore, in the future study, the negative emotion should be taken into account.

Finally, Čeněk and Šašinka (2015) believe that there are cultural based differences between visual perception and related cognitive processes on attention and memory. According to their research, East Asians and Westerners perceive and think about objects differently. The Westerners are prone to pay attention to some focus objects (the size, movement and colors of the object) to and analyze its attributes. However, East Asians are more preference to focusing on a wide range of perception areas and notice relationships and changes of the objects. Hence, the incomplete face of spokes-characters in icons of our work will bring higher anthropomorphic perception and stronger interpersonal closeness, which may only be apply to eastern participants, and this effect may not exist for western participants.

## Data Availability Statement

The datasets generated for this study are available on request to the corresponding author.

## Ethics Statement

Ethical review and approval was not required for the study on human participants in accordance with the local legislation and institutional requirements. Written informed consent from the participants’ legal guardian/next of kin was not required to participate in this study in accordance with the national legislation and the institutional requirements.

## Author Contributions

ZNi, LC, HY, and TZ contributed conception and design of the study. ZNi organized the database. LC performed the statistical analysis. LC and HY wrote the first draft of the manuscript. ZNi, ZNa, LC, TZ, and HY wrote sections of the manuscript. All authors contributed to manuscript revision, read and approved the submitted version.

## Conflict of Interest

The authors declare that the research was conducted in the absence of any commercial or financial relationships that could be construed as a potential conflict of interest.

## Footnotes

1 https://www.sojump.com/

2 https://www.sojump.com/

## References

[B1] Aaker J. L. (1997). Dimensions of brand personality. *J. Mark. Res.* 34 347–356.

[B2] Aaker J. Fournier S. Brasel S. A. (2004). When good brands do bad. *J. Consum. Res.* 31 1–6.

[B3] Aggarwal P. Mcgill A. (2007). Is that car smiling at me? schema congruity as a basis for evaluating anthropomorphized products. *J. Consum. Res.* 34 468–479. 10.1086/518544

[B4] Aggarwal P. Mcgill A. L. (2012). When brands seem human, do humans act like brands? automatic behavioral priming effects of brand anthropomorphism. *J. Consum. Res.* 39 307–323. 10.1086/662614

[B5] Aknin L. B. Broesch T. Hamlin J. K. Van J. W. (2015). Pro-social behavior leads to happiness in a small-scale rural society. *J. Exper. Psychology* 144 788–795. 10.1037/xge0000082 26030168

[B6] Aron A. Norman C. C. Aron E. N. Mckenna C. Heyman R. E. (2000). Couples’ shared participation in novel and arousing activities and experienced relationship quality. *J. Person Soc. Psychol.* 78 273–284. 10.1037/0022-3514.78.2.273 10707334

[B7] Barbara J. P. (1996). Defining trade characters and their role in american popular culture. *J. Pop. Cult.* 29 143–158. 10.1111/j.0022-3840.1996.1438797.x

[B8] Baumeister R. F. Dewall C. N. Ciarocco N. J. Twenge J. M. (2005). Social exclusion impairs self-regulation. *J. Person Soc. Psychol.* 88 589–604. 10.1037/0022-3514.88.4.589 15796662

[B9] Baumeister R. F. Leary M. R. (1995). The need to belong: desire for interpersonal attachments as a fundamental human motivation. *Psychol. Bull.* 117 497–529. 10.1037/0033-2909.117.3.497 7777651

[B10] Burgoon J. K. Hale J. L. (1987). Validation and measurement of the fundamental themes of relational communication. *Commun. Monogr.* 54 19–41. 10.1080/03637758709390214

[B11] Callcott M. F. Alvey P. A. (1991). “Toons sell and sometimes they don’t: An advertising spokes-character typology and exploratory study,” in *Proceedings of the 1991 conference of the American Academy of Advertising* (New York, NY: American Academy of Advertising).

[B12] Callcott M. F. Phillips B. J. (1996). Observations: elves make good cookies: creating likable spokes-character advertising. *J. Advert. Res.* 36 73–78.

[B13] Calvert S. L. (2008). Children as consumers: advertising and marketing. *Fut. Children* 18 205–234. 10.1353/foc.0.0001 21338011

[B14] Čeněk J. Šašinka È (2015). Cross-cultural differences in visual perception[J]. *J. Educ. Cult. Soc.* 2015 187–206. 10.15503/jecs20151.187.206

[B15] Chiu Y. Lin C. Liu W. (2009). The affect transfer effect on spokes-character. *ACME Proc.* 386–398.

[B16] En-Chi C. (2014). Influences of the spokes-character on brand equity antecedents. *Asia Pacific J. Mark. Logistics* 26 494–515. 10.1108/apjml-02-2013-0030

[B17] Cian L. Krishna A. Elder R. (2014). This logo moves me: dynamic imagery from static images. *Soc. Sci. Electr. Publ.* 51 184–197. 10.1509/jmr.13.0023 11670861

[B18] Clary S, M. Ridge R. D. Copeland J. Stukas A. A. Haugen J. (1998). Understanding and assessing the motivations of volunteers: a functional approach. *J. Person Soc. Psychol.* 74 1516–1530. 10.1037/0022-3514.74.6.1516 9654757

[B19] Delbaere M. Mcquarrie E. F. Phillips B. J. (2011). Personification in advertising using a visual metaphor to trigger anthropomorphism. *J. Advert.* 40 121–130. 10.2753/joa0091-3367400108

[B20] Dewall C. N. Schaller M. Maner J. K. Baumeister R. F. (2007). Does social exclusion motivate interpersonal reconnection? resolving the “porcupine problem. *J. Person Soc. Psychol.* 92 42–55. 10.1037/0022-3514.92.1.42 17201541

[B21] Epley N. Waytz A. Akalis S. Cacioppo J. T. (2008). When we need a human: motivational determinants of anthropomorphism. *Soc. Cogn.* 26 143–155. 10.1521/soco.2008.26.2.143

[B22] Epley N. Waytz A. Cacioppo J. T. (2007). On seeing human: a three-factor theory of anthropomorphism. *Psychol. Rev.* 114 864–886. 10.1037/0033-295x.114.4.864 17907867

[B23] Eppler M. J. Burkard R. A. (2004). *Knowledge Visualization: Towards a New Discipline and its Fields of Application, ICA Working Paper.* Schwartz: University of lugano.

[B24] Folse J. A. G. Burton S. (2012). Spokes-characters: how the personality traits of sincerity, excitement, and competence help to build equity. *J. Advert.* 41 17–32.

[B25] Folse J. A. G. Burton S. Netemeyer R. C.. (2013). Defending brands: effects of alignment of spokescharacter personality traits and corporate transgressions on brand trust and attitudes. *J. Advert.* 42 331–342. 10.1080/00913367.2013.795124

[B26] Garretson J. A. Burton S. (2005). The role of spokes-characters as advertisement and package cues in integrated marketing communications. *J. Mark.* 69 118–132. 10.1509/jmkg.2005.69.4.118 11670861

[B27] Guthrie S. E. (1993). *Faces in the Clouds.* Oxford: Oxford University Press.

[B28] Hagtvedt H. (2011). The impact of incomplete typeface logos on perceptions of the firm. *J. Mark.* 75 86–93. 10.1509/jmkg.75.4.86 11670861

[B29] Hayes A. (2013). Introduction to mediation, moderation, and conditional process analysis. *J. Educ. Meas.* 51 335–337. 10.1111/jedm.12050

[B30] Henderson P. W. Cote J. A. (1998). Guidelines for selecting or modifying logos. *J. Mark.* 62 14–30. 10.1177/002224299806200202

[B31] Hur J. D. Koo M. Hofmann W. (2015). When temptations come alive: how anthropomorphism undermines self-contro. *J. Consum. Res.* 42 340–358.

[B32] Lagerwerf L. Hooijdonk C. M. J. Ayalies K. (2012). Processing visual rhetoric in advertisements: interpretations determined by verbal anchoring and visual structure. *J. Pragm.* 44 1836–1852. 10.1016/j.pragma.2012.08.009

[B33] Lakoff G. Mark J. (1980). *Metaphors We Live By.* Chicago, IL: University of Chicago Press.

[B34] Leary M. R. Kowalski R. M. Smith L. Phillips S. (2003). Teasing, rejection, and violence: case studies of the school shootings. *Aggres. Behav.* 29 202–214. 10.1002/ab.10061

[B35] Lee A. Y. Labroo A. A. (2004). The effect of conceptual and perceptual fluency on brand evaluation. *J. Mark. Res.* 41 151–165. 10.1509/jmkr.41.2.151.28665 11670861

[B36] Lee J. Shrum L. J. (2012). Conspicuous consumption versus charitable behavior in response to social exclusion: a differential needs explanation. *J. Consum. Res.* 39 530–544. 10.1086/664039

[B37] Lewis M. (2000). “Self-conscious emotions: embarrassment, pride, shame, and guilt,” in *Handbook of Emotions*, eds Lewis M. Haviland-Jones J. (New York, NY: Guilford Press), 623–636.

[B38] Mazzini P. Almici M. Bottazzi L. A. Magri M. Sommese G. (2011). *L’inclusione Sociale tra Gli Adolescenti: Una Sfida Possibile?. Cognitivismo Clinico.* Rome: Giovanni Fioriti Editore Srl.

[B39] Miller E. Kahn B. (2005). Shades of meaning: the effect of color and flavor names on consumer choic. *J. Consum. Res.* 32 86–92. 10.1086/429602

[B40] Molden D. C. Lucas G. M. Gardner W. L. Dean K. Knowles M. L. (2009). Motivations for prevention or promotion following social exclusion: being rejected versus being ignored. *J. Person Soc. Psychol.* 96 415–431. 10.1037/a0012958 19159140

[B41] Morewedge C. K. Jesse P. Daniel M. W. (2004). *Timescale Anthropomorphism in the Attribution of Mind. Working paper.* Bostonm, MA: Harvard University.

[B42] Mulken M. V. Hooft A. V. Nederstigt U. (2014). Finding the tipping point: visual metaphor and conceptual complexity in advertising. *J. Advert.* 43 333–343. 10.1080/00913367.2014.920283

[B43] Nazuk S. Sajeev V. (2018). Active white space (AWS) in logo designs: effects on logo evaluations and brand communication. *J. Advert.* 47 1–12.

[B44] Olsen G. Douglas J. W. P. Thomas C. O. G. (2012). Print advertising: white space. *J.Bus. Res.* 65 855–860. 10.1016/j.jbusres.2011.01.007

[B45] Orehek E. Forest A. L. Wingrove S. (2018). People as means to multiple goals: implications for interpersonal relationships. *Pers. Soc. Psychol. Bull.* 44 1487–1501. 10.1177/0146167218769869 29742998

[B46] Peracchio L. A. Meyers-Levy J. (1994). How ambiguous cropped objects in ad photos can affect product evaluations. *J. Consum. Res.* 21:190 10.1086/209392

[B47] Phillips J. D. (1996). Aesthetic mathematics: does mathematics have to be justified by its. *Austr. Math. Teacher.* 52 14–18.

[B48] Pieters R. Wedel M. Batra R. (2010). The stopping power of advertising: measures and effects of visual complexity. *J. Mark.* 74 48–60. 10.1509/jmkg.74.5.048 11670861

[B49] Poggi I. D’Errico F. (2010a). The mental ingredients of bitterness. *J. Mult. Interfaces* 3 79–86. 10.1007/s12193-009-0021-9

[B50] Poggi I. D’Errico F. (2010b). *Cognitive modelling of human social signals[C]. International Workshop on Social Signal Processing.* New Yok, NY: ACM.

[B51] Poggi I. Zuccaro V. (2008). “Admiration,” in *Proceedings of the AFFINE workshop.*

[B52] Pracejus J. Olsen G. O’Guinn Thomas C. (2006). How nothing became something: white space, rhetoric, history, and meaning. *J. Consum. Res.* 33 82–90. 10.1086/504138

[B53] Puzakova M. Aggarwal P. (2018). Brands as rivals: consumer pursuit of distinctiveness and the role of brand anthropomorphism. *J. Consum. Res.* 45 869–888. 10.1093/jcr/ucy035

[B54] Puzakova M. Kwak H. (2017). Should anthropomorphized brands engage customers? the impact of social crowding on brand preferences. *J. Market.* 81 99–115. 10.1509/jm.16.0211 11670861

[B55] Puzakova M. Kwak H. Rocereto J. F. (2013). When humanizing brands goes wrong: the detrimental effect of brand anthropomorphization amid product wrongdoings. *J. Mark.* 77 81–100. 10.1509/jm.11.0510 11670861

[B56] Refaie E. (2003). Understanding visual metaphor: the example of newspaper cartoons. *Vis. Commun.* 2 75–95. 10.1177/1470357203002001755

[B57] Rizzi E. R. (2007). *Itinerari Del Rancore.* Torino: Bollati Boringhieri.

[B58] Schilperoord J. Maes A. Ferdinandusse H. (2009). Perceptual and conceptual visual rhetoric: the case of symmetric object alignment. *Metap. Symbol* 24 155–173. 10.1080/10926480903028110

[B59] Schroll R. Schnurr B. Grewal D. (2018). Humanizing products with handwritten typefaces. *J. Consum. Res.* 45 648–672.

[B60] Sinha J. Fang-Chi L. (2019). Ignored or rejected: retail exclusion effects on construal levels and consumer responses to compensation. *J. Consum. Res.* 46 791–807. 10.1093/jcr/ucz021

[B61] Su L. Jiang Y. Chen Z. Nathan D. C. (2017). Social exclusion and consumer switching behavior: a control restoration mechanism. *J. Consum. Res.* 44 99–117. 10.1093/jcr/ucw075

[B62] Tremoulet P. D. Feldman J. (2000). Perception of animacy from the motion of a single object. *Perception* 29 943–951. 10.1068/p3101 11145086

[B63] Twenge J. M. Baumeister R. F. Dewall C. N. Ciarocco N. J. Bartels J. M. (2007). Social exclusion decreases prosocial behavior. *J. Person Soc. Psychol.* 92 56–66. 10.1037/0022-3514.92.1.56 17201542

[B64] Twenge J. M. Baumeister R. F. Tice D. M. Stucke T. S. (2001). If you can’t join them, beat them: effects of social exclusion on aggressive behavior. *J. Person Soc. Psychol.* 81 1058–1069. 10.1037/0022-3514.81.6.1058 11761307

[B65] Twenge J. M. Catanese K. R. Baumeister R. F. (2002). Social exclusion causes self-defeating behavior. *J. Person Soc. Psychol.* 83 606–615. 10.1037/0022-3514.83.3.606 12219857

[B66] Vinciarelli A. Pantic M. Heylen D. Pelachaud C. Poggi I. D’Errico F. (2011). Bridging the gap between social animal and unsocial machine: a survey of social signal processing. *IEEE Trans. Affect. Computing* 3 69–87. 10.1109/t-affc.2011.27

[B67] Waytz A. Morewedge C. K. Epley N. Monteleone G. Gao J.-H. Cacioppo J. T. (2010). Making sense by making sentient: effectance motivation increases anthropomorphism. *J. Pers. Soc. Psychol.* 99 410–435. 10.1037/a0020240 20649365

[B68] Wertheimer M. (1938). *Laws of Organization in Perceptual Forms. A Source Book of Gestalt Psychology.* Abingdon: Routledge & Kegan Paul, 71–88.

[B69] Williams K. D. (2007). Ostracism. *Ann. Rev. Psychol.* 58 425–452. 16968209 10.1146/annurev.psych.58.110405.085641

[B70] Woosnam K. M. (2010). The inclusion of other in the self (IOS) scale. *Ann. Tour. Res.* 37 857–860. 10.1016/j.annals.2010.03.003

[B71] Zeelenberg M. Beattie J. Van der Pligt J. Vries N. K. (1996). Consequences of regret aversion: effects of expected feedback on risky decision making. *Organ. Behav. Hum. Dec. Proc*. 65 148–158. 10.1006/obhd.1996.0013

[B72] Zhao S. H. Meyer R. J. (2007). Biases in predicting preferences for the whole visual patterns from product fragments. *J. Consum. Psychol.* 17 292–304. 10.1016/s1057-7408(07)70039-6

[B73] Zhong D. Zhang J. P. (2009). Knowledge visualization ——an approach of knowledge transfer and restructuring in education. *Int. For. Inform. Technol. Appl.* 2009 716–719.

