# Supplementary material for: The Impact of Incomplete Faces of Spokes-Characters in Mobile Application Icon Designs on Brand Evaluations
Source: Front Psychol. 2020 Jul 31;11:1495. doi: 10.3389/fpsyg.2020.01495 (PMC7411004; doi:10.3389/fpsyg.2020.01495)
Supplement: Supplementary file 1 [file Table_1.DOC]

**Appendix A**


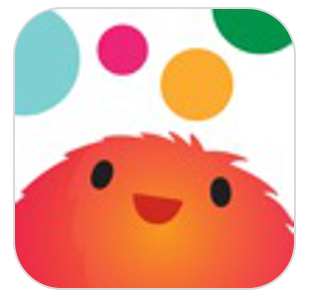

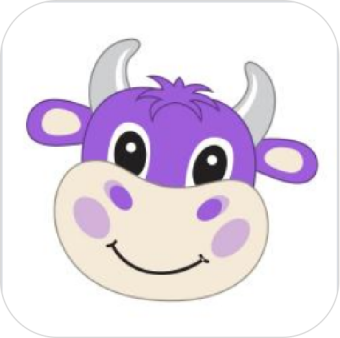


**Hopster**

**（ the incomptele face of spokes-character）**

**Happy Cow Find Vegan Food**

**（ the comptele face of spokes-character）**

**Appendix B**


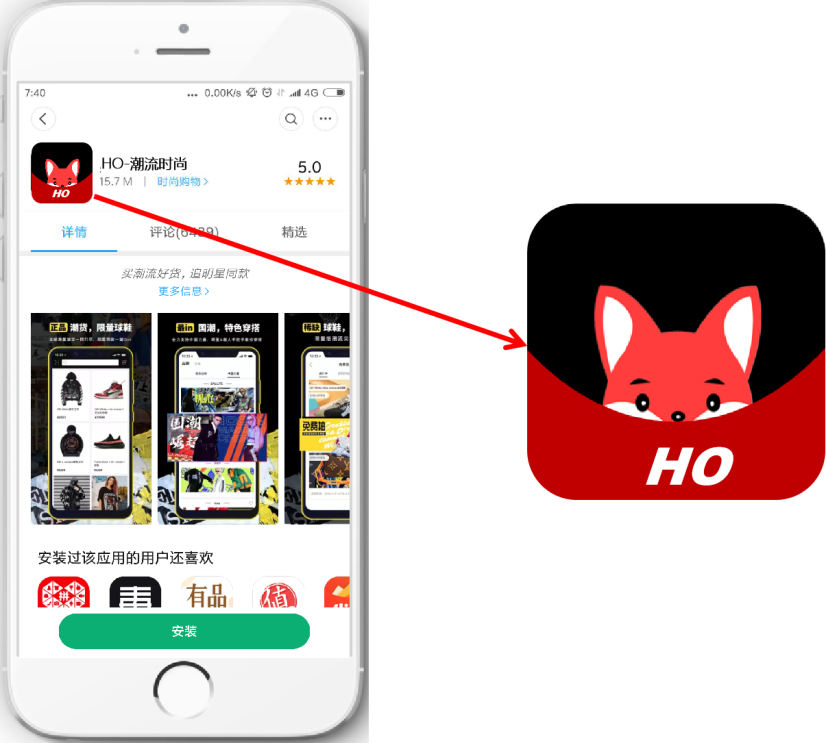


**Incomplete**

**
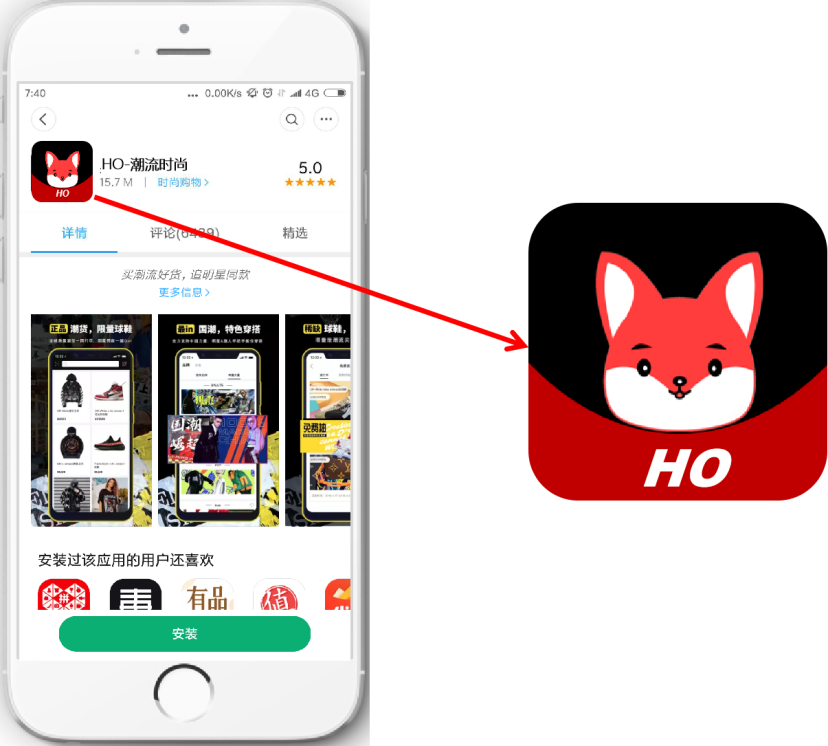
**

**Complete**

Note: the translations of main Chinese characters in the picture above 1) 潮流时尚：Fashion；2）详情：Details；3）评论：Comments；4）精选：Carefully Selected；5）时尚购物：Fashion Shopping；6)安装：Install

**Appendix C**

**Incomplete**


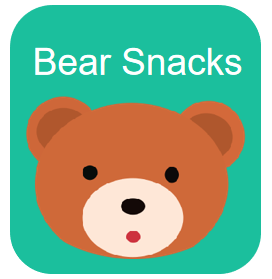

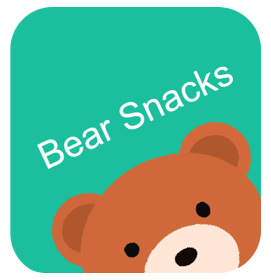


**Complete**

**Appendix D**

We conducted a separate experiment to rule out the mediation of the interesting (playfulness) and interactivity. An incomplete (vs. complete) typeface logo causes consumers to perceive the firm as more (vs. less) creative and innovative. And this influence is mediated by the perceived interestingness of the logo (Hagtvedt, 2011). In addition, a virtual character could convey the social signal (Poggi et al., 2014). Hence, the spoke-character might induce more perception of interactivity.

In this study, we investigated whether the use of an incomplete (vs. complete) spokes-character face in a launch icon enhanced interesting and interactivity of the spokes-character to ultimately and lead to more favorable brand evaluations.

***1 Design***

We employed a single-factor (completeness: incomplete vs. complete) between-subjects design. One hundred thirty participants with various backgrounds were recruited from Sojump (https://www.sojump.com/). Three participants who failed to complete the survey were excluded from the final analyses. The remaining 128 participants provided complete datasets (77 females; M_age_= 28.34, SD_age_ = 6.88). We used the icon of a mobile application for a fictitious snack brand (Bear Snack) as our stimulus to extend our findings (see appendix B).

***2 Procedures***

Firstly, we introduced the fictitious snack brand (a chain of casual snacks brand, which optimizes a variety of domestic snacks, such as pastries, candy, preserves and other products) and then we present the icon. After that, the participants answered the same manipulation item and evaluate the brand evaluations as in study 1. Next, we measured perceptions of interesting (Interesting/ Not Interesting; Not boring/ Boring; Makes me curious/ Does not make me curious; Keeps my attention/ Does not keep my attention (1 = “not at all” and 7 = “very much”) (α= .83; Lee, Tinkham and Edwards, 2005) and interactivity with three items adopted from prior research on interpersonal evaluations with three items (“I perceive that I am in the presence of the spokes-character”; “ I feel that the spokes-character is watching me and is aware of my presence”; “The spokes-character appears to be sentient (conscious and alive) to me” (α=.79) (Blascovich et al., 2002) . Finally, the participants provided brief demographic information.

***3 Results and Discussion***

*Manipulation check.* We conducted a manipulation check on facial completeness and demonstrated that users in the complete face condition perceived the facial completeness of the spokes-character to be more complete than those in the incomplete face condition (M_C_ = 5.78, SD=1.19; M_IC_ = 2.65, SD=1.44; *F*(1, 76) = 76.32, *p* < .001).

*Brand evaluations.* We analyzed our predictions using a one-factor (completeness: incomplete vs. complete) ANOVA with brand evaluations as the dependent variable. The results revealed significant differences in brand evaluations (M_IC_ = 5.60, SD=1.07; M_C_ = 4.59, SD=1.42; *F*(1, 77) = 15.23, *p* < 0.001), which supported H1 again.

*Mediation analyses.* To further examine the underlying mechanism of the effect of an incomplete spokes-character face in an icon design, we tested regression models with incomplete spokes-character face, perceptions of interesting, and interactivity as the mediators respectively and brand evaluations as the dependent variable (Hayes 2013, Model 4) using a bootstrapping approach. We found that perceptions of interesting (95% CI [-0.0212,0.1222]), and interactivity do not mediate the effect of incomplete spokes-character face on brand evaluations (95% CI [-0.0163,0.2093]).

The results indicated that the positive impact of an incomplete spokes-character face is not driven by perceptions of interesting or interactivity. So we rule out the two possible mediators.
